# Phagocytosis and Epithelial Cell Invasion by Crohn’s Disease-Associated Adherent-Invasive *Escherichia coli* Are Inhibited by the Anti-inflammatory Drug 6-Mercaptopurine

**DOI:** 10.3389/fmicb.2018.00964

**Published:** 2018-05-14

**Authors:** Federica Migliore, Raffaella Macchi, Paolo Landini, Moira Paroni

**Affiliations:** Dipartimento di Bioscienze, Università degli studi di Milano, Milan, Italy

**Keywords:** adherent-invasive *E. coli*, Crohn’s disease, azathioprine, 6-mercaptopurines, c-di-GMP, dysbiosis, virulence factors

## Abstract

Adherent-invasive *Escherichia coli* (AIEC) strains are overrepresented in the dysbiotic microbiota of Crohn’s disease (CD) patients, and contribute to the onset of the chronic inflammation typical of the disease. However, the effects of anti-inflammatory drugs used for CD treatment on AIEC virulence have not yet been investigated. In this report, we show that exposure of AIEC LF82 strain to amino-6-mercaptopurine (6-MP) riboside, one of the most widely used anti-inflammatory drugs in CD, impairs its ability to adhere to, and consequently to invade, human epithelial cells. Notably, phagocytosis of LF82 treated with 6-MP by human macrophages is also reduced, suggesting that 6-MP affects AIEC cell surface determinants involved both in interaction with epithelial cells and in uptake by macrophages. Since a main target of 6-MP in bacterial cells is the inhibition of the important signal molecule c-di-GMP, we also tested whether perturbations in cAMP, another major signaling pathway in *E. coli*, might have similar effects on interactions with human cells. To this aim, we grew LF82 in the presence of glucose, which leads to inhibition of cAMP synthesis. Growth in glucose-supplemented medium resulted in a reduction in AIEC adhesion to epithelial cells and uptake by macrophages. Consistent with these results, both 6-MP and glucose can affect expression of cell adhesion-related genes, such as the *csg* genes, encoding thin aggregative fimbriae (curli). In addition, glucose strongly inhibits expression of the *fim* operon, encoding type 1 pili, a known AIEC determinant for adhesion to human cells. To further investigate whether 6-MP can indeed inhibit c-di-GMP signaling in AIEC, we performed biofilm and motility assays and determination of extracellular polysaccharides. 6-MP clearly affected biofilm formation and cellulose production, but also, unexpectedly, reduced cell motility, itself an important virulence factor for AIEC. Our results provide strong evidence that 6-MP can affect AIEC-host cell interaction by acting on the bacterial cell, thus strengthening the hypothesis that mercaptopurines might promote CD remission also by affecting gut microbiota composition and/or physiology, and suggesting that novel drugs targeting bacterial virulence and signaling might be effective in preventing chronic inflammation in CD.

## Introduction

Crohn’s disease (CD), a type of inflammatory bowel disease (IBD), is characterized by chronic and relapsing intestinal inflammation resulting from inappropriate and persistent activation of the mucosal immune system (de Souza and Fiocchi, 2016). The pathophysiology of CD is determined by a variety of elements, including heritable traits (Duerr et al., 2006; Villani et al., 2009), environmental factors (Ng et al., 2013), abnormalities in the intestinal mucosal barrier (Wang et al., 2016), and aberrant immune response (Geremia et al., 2014). However, there is increasing evidence pointing to dysbiosis, i.e., an altered composition of the normal gut microbiota resulting in a breakdown of host–microbial mutualism, as a key factor in CD pathogenesis (Ahmed et al., 2016). Indeed, the shift from predominant symbiont microorganisms to potential harmful “pathobiont” microbes has been well documented in IBD by various clinical studies (Frank et al., 2007; Kaur et al., 2011). Metagenomic analyses of human gut microbiota in IBD patients have also revealed hallmark shifts in microbial abundances in comparison to healthy individuals, characterized in particular by an enrichment in pathobionts from Bacteroidetes and enterobacteria and a concomitant depletion of symbionts including Firmicutes, Bifidobacteria, and Clostridia (Walters et al., 2014; Gevers et al., 2017). Conversely, for other bacterial genera, results from metagenomic analyses comparing healthy subjects and CD patients have not always provided consistent results, due for instance to the comparison of very diverse samples such as human fecal rather than mucosal microbiota (Zoetendal et al., 2002). However, despite some differences, the whole of metagenomic data points to the importance of a relatively small group of pathogenic microorganisms in setting off the onset of CD (Bull et al., 2003; Laharie et al., 2009). In particular, adherent-invasive *Escherichia coli* (AIEC) are enriched in ileal specimens from CD patients, in comparison to non-IBD patients; remarkably, however, AIEC were rarely found in samples from ulcerative colitis (UC) patients (Boudeau et al., 1999; Darfeuille-Michaud et al., 2004; Martinez-Medina et al., 2009). The important role played by AIEC in CD pathogenicity is due to their ability to invade both intestinal epithelial cells and macrophages, in turn resulting in high levels of secretion of pro-inflammatory cytokines ultimately contributing to chronic inflammation (Glasser et al., 2001; Eaves-Pyles et al., 2008).

6-Mercaptopurines (6-MP) have been used for several decades as anti-inflammatory drugs in IBD treatment, to counteract chronic inflammation (Lennard, 1992). Azathioprine, the 6-MP drug most commonly employed in therapy, is metabolically activated and converted into thioguanine nucleotides (TGN), which are ultimately responsible for its anti-inflammatory activity. In particular, azathioprine metabolite 6-thioguanosine-triphosphate (6-T-GTP) has been shown to prevent Rac1 and Rac2 activation in human CD4^+^ lymphocytes blocking T-cell activation (Tiede et al., 2003), and also to inhibit Rac1 in macrophages and intestinal epithelial cells, thus reducing their inflammation and proliferation (Marinkovic et al., 2014). However, in addition to its activity on the immune system cells, azathioprine also shows some antibacterial activity, for instance against *Mycobacterium avium* subspecies *paratuberculosis* (MAP), the etiological agent of chronic granulomatous enteritis in cattle (Johne’s disease), which bears some resemblance to human CD (Shin and Collins, 2008). Interestingly, several published studies have indicated that MAP, similarly to AIEC, is present in higher amounts in CD patients than in UC patients and non-inflammatory IBD (Feller et al., 2007; Abubakar et al., 2008) suggesting a possible important role for these bacteria in CD immunopathology. In a previous work, we showed that azathioprine can inhibit biosynthesis of the signal molecule c-di-GMP in the *E. coli* MG1655 laboratory strain, possibly via inhibition of *de novo* purine biosynthesis (Antoniani et al., 2013). c-di-GMP controls the synthesis of various highly antigenic extracellular structures in bacteria (Cotter and Stibitz, 2007), and is also a powerful effector of the human immune response (Karaolis et al., 2007; Gray et al., 2012), suggesting that inhibition of c-di-GMP synthesis in bacteria might tone down its immunostimulatory effect, relieving inflammation. Altogether, these observations suggest that the anti-inflammatory activity of 6-MP might target not only human macrophages, epithelial cells, and lymphocytes, but also the gut microbiota, by impacting either growth or physiological conditions (or both) of bacteria linked to CD. However, the direct effect of 6-MP on AIEC-virulence factors has never been characterized.

In this work, we show that the AIEC strain LF82, grown in the presence of amino-6-MP riboside, a metabolite of azathioprine, is affected in its ability to attach and, consequently to invade human epithelial cells. We also analyzed 6-MP effects on AIEC virulence in the presence of glucose, and those of glucose alone. Indeed, in the ileal mucosa, i.e., the site where AIEC is found with higher frequency in CD patients, glucose concentrations can vary considerably, reaching higher concentrations than in the bloodstream (Holst et al., 2016). Transient exposure to glucose might modulate production of AIEC virulence determinants, as reported for other pathogens (Munson, 2013; Daddaoua et al., 2014; Rossi et al., 2016). Finally, while 6-MP is an inhibitor of c-di-GMP, glucose strongly represses cAMP production in *E. coli*, thus affecting another important nucleotide-derived signal molecule. We found that pre-growth of AIEC in glucose-supplemented medium impaired adhesion to epithelial cells and uptake by macrophages, to an extent similar to 6-MP, likely via inhibition of cAMP biosynthesis. Our results strongly suggest that targeting bacterial–host interaction might represent an important strategy for the development of novel therapeutics aimed at the remission and treatment of CD.

## Materials and Methods

### Bacterial Strains, Plasmids, and Growth Conditions

The LF82 strain used in this study was originally isolated from an ileal biopsy specimen from a CD patient (Boudeau et al., 1999). As a non-AIEC control, the standard laboratory strain MG1655 (Blattner et al., 1997) was used in our experiments. Bacteria were growth either in YESCA medium (10 g/l casamino acids, 1.5 g/l yeast extract) or in YESCA medium supplemented with 0.2% (11.1 mM) glucose (YESCA-GLU). 6-MP was added at either 2 μg/ml (6.7 μM) to liquid cultures or 8 μg/ml (26.7 μM) to cultures grown on solid medium. These concentrations are subinhibitory for growth in the different conditions, as the minimal inhibitory concentration (MIC) of 6-MP in YESCA liquid medium is 64 μg/ml (213 μM) (Supplementary Figure 1). In order to perturb intracellular c-di-GMP concentrations, LF82 was transformed either with pTOPOAdrA (referred to as pAdrA from now on) (Gualdi et al., 2008), for the overexpression of the diguanylate cyclase-encoding *adrA* gene (Zogaj et al., 2001), or with the pTOPODosP, to overexpress the *dosP* gene, coding for a c-di-GMP phosphodiesterase (referred to as pDosP from now on) (Schmidt et al., 2005), or with the control vector pTOPO (pCR2.1-TOPO, Invitrogen). For plasmid-bearing strains, growth media was always supplemented with 50 μg/ml kanamycin.

### Generation of Human Monocytes-Derived Macrophages

Human monocytes were purified from buffy coat of healthy donors (obtained from Fondazione IRCCS Ca’ Granda Ospedale Maggiore Policlinico in Milan), by Ficoll density gradient separation, and by positive selection using CD14^+^ selection (CD14 Microbeads, Miltenyi Biotec). Monocytes were suspended in complete RPMI medium (10% heat-inactivated FCS, 1 mmol/l sodium pyruvate, 10 mmol/l non-essential amino acids, and 1% penicillin/streptomycin) supplemented with 0.2 μg/ml of recombinant human macrophage colony stimulating factor (rh-M-CSF, Miltenyi Biotec). CD14^+^ cells were seeded into 24-well culture plates at a density of 2 × 10^5^ and were incubated at 37°C in a humidified 5% CO_2_ atmosphere for 6 days.

### Phagocytosis Assay

Efficiency of either LF82 or MG1655 phagocytosis by human macrophages was determined using the gentamicin protection assay (Glasser et al., 2001). Prior to addition of bacteria, monocytes-derived macrophage (MDM) monolayers were washed twice with 1× PBS, and the medium was replaced with 1 ml of complete RPMI medium without penicillin/streptomycin. Bacteria, grown overnight in different conditions, were washed and resuspended in complete RPMI medium without penicillin/streptomycin. MDM were infected at a multiplicity of infection (MOI) of 100 bacteria per macrophage. After a 10 min centrifugation at 1,000 *g*, monocytes were incubated 1 h at 37°C to allow internalization. Cell monolayers were washed twice in PBS to remove extracellular bacteria and treated with RPMI containing 20 μg/ml gentamicin (20-fold the MIC for both LF82 and MG1655) for 1 h to kill non-internalized bacteria. Subsequently, the monolayers were washed twice with PBS, and cells were lysed by adding deionized water containing 1% (vol/vol) Triton X-100 (Sigma) for 10 min, to release internalized bacteria. To obtain a precise determination of the bacterial titer, cell lysates were serially diluted, 100 μl from each dilution was plated on LB agar plates, and CFU were determined after 24 h growth at 37°C by viable count.

### Adherence and Invasion Assays

Adhesion and invasion of epithelial cells by bacterial strains MG1655 and LF82, grown in different conditions, were determined using the intestinal cell line HT29 as previously described (Martin et al., 2004). HT29 cells were seeded into 24-well tissue culture plates at 4 × 10^5^ cells per well and maintained in complete RPMI medium until a confluent monolayer was formed. Bacterial cells, grown in different conditions, were washed and resuspended in complete RPMI medium without penicillin/streptomycin, and used, at 7 × 10^6^ bacteria/well, to infect monolayers, which had previously been washed twice with sterile PBS. Incubation was carried out for 3 h at 37°C in complete medium without penicillin/streptomycin in a humidified 5% CO_2_ atmosphere. To determine the total number of cell-associated bacteria, corresponding to adherent and intracellular bacteria, epithelial monolayers were washed three times with sterile PBS to remove unattached bacteria and then lysed with deionized water containing 1% (vol/vol) Triton X-100. To determine bacterial invasion, epithelial monolayers, after being incubated 3 h with bacteria and washed three times with PBS, were incubated for 1 h in cell culture medium containing 20 μg/ml of gentamicin, to kill extracellular bacteria and washed again with PBS. The intestinal epithelial cells were then lysed with 1% Triton X-100 in deionized water. The amount of intracellular bacteria recovered from the lysed monolayers was quantified as described for the phagocytosis assay.

### Immunofluorescence and Confocal Microscopy

HT29 cell monolayers on glass coverslips were infected with LF82 cultures, grown overnight in different conditions, washed and resuspended as previously described. After 3 h incubation in RPMI medium, cells were washed, fixed in 4% paraformaldehyde (PF)/PBS, blocked in 2% FBS/PBS and stained with rabbit anti-*E. coli* antibody (ab20640, Abcam 1:100) for 1 h. Coverslips were then incubated with goat anti-rabbit Alexa Fluor 488 diluted 1:500 (Invitrogen). Nuclei were stained with 4′,6-diamidino-2-phenylindole (DAPI, Molecular Probes). Images were acquired by a Nikon A1 laser scanning confocal microscopy using a 60× oil immersion objective (NA 1.4). The Z-stacks were acquired taking an image every 0.5 μm.

### Outer Membrane Protein Isolation

Cell fractionation was performed as described previously (Deflaun et al., 1994). Briefly, 250 ml cultures, grown in different conditions for 18 h, were centrifuged at 4,000 *g* for 10 min at 4°C and washed with 5 ml 0.1 M phosphate buffer (PB) pH 7.0. Cells were resuspended in 2 ml PB with addition of 100 μg/ml lysozyme and 1 mM EDTA pH 8.0 and incubated at room temperature for 10 min. Cells were disintegrated using a French Press and centrifuged as above to remove unbroken cells. The low-speed centrifugation supernatant was then centrifuged at 100,000 *g* for 1 h at 4°C to separate the cytoplasm (supernatant) and the membrane fraction (pellet). The pellet was resuspended in 2 ml of 2% Sarkosyl in PB, left for 20 min at room temperature, and centrifuged at 40,000 *g* at 10°C for 10 min to remove ribosomes and cytoplasmic proteins that were still associated with the membrane fraction. The pellet was resuspended in 1 ml of 1% Sarkosyl, precipitated again for 20 min at room temperature, and centrifuged as before, and the pellet, corresponding to outer membrane proteins, was resuspended in 0.5 ml H_2_O. Protein concentrations were determined using standard Bradford assay, and 10 μg total proteins was loaded onto a 12% sodium dodecyl sulfate-polyacrylamide gel (SDS-PAGE). Specific bands were identified by matrix assisted laser desorption/ionization-time of flight (MALDI-TOF) analysis of the peptide products after in-gel trypsin digestion (Chen et al., 2000); performed by CEINGE, University of Naples, Italy^1^.

### Yeast Cell Aggregation Assay

Yeast aggregation was determined as described (Claret et al., 2007). Commercial baker’s yeast (*Saccharomyces cerevisiae*) was suspended in PBS (10 mg, dry weight/ml). Overnight bacterial cultures, grown in various conditions, were resuspended in PBS to an OD_600_ = 0.3. Equal volumes of fixed yeast cell suspension and decreasing concentrations of *E. coli* suspension were mixed in a 96-well plate. Aggregation was monitored visually, and the titer was recorded as the last dilution of bacteria giving a positive aggregation reaction.

### Transmission Electron Microscopy

Overnight bacterial cultures, grown in various conditions, were fixed and negatively stained with 1% ammonium molybdate on carbon-Formvar copper grids as described (Claret et al., 2007).

### Gene Expression Determination by Real-Time PCR

For RNA isolation, strains were grown in various conditions either at 28°C or at 37°C; for each gene tested, we determined the timing of its maximal expression, choosing from: mid log, onset of stationary phase (termed as “late log” in the figures), or late stationary phase (Supplementary Figure 4 and **Figure 4A**). Bacterial cells were harvested by centrifugation at 10,000 *g* for 5 min at 4°C, and total RNA was extracted using RNeasy Mini Kit (QIAGEN). RNA samples were checked by agarose gel electrophoresis to assess lack of degradation, and quantified spectrophotometrically. Genomic DNA was removed by DNase I treatment. Reverse transcription was performed on 1 μg total RNA, along with negative control samples incubated without reverse transcriptase. cDNA synthesis efficiency was verified by electrophoresis on agarose gel in comparison to negative controls. Real-time PCR was performed using the SYBR Green PCR master mixture, and the results were determined with an iCycler iQ Real-Time detection system (Bio-Rad). Reaction mixtures (25 μl) included 0.1 μg cDNA and 300 nM primers in the reaction buffer and enzyme supplied by the manufacturer. Primer sequences are listed in Supplementary Table 1. All reactions were performed in duplicate, including negative control samples, which never showed significant threshold cycles. A minimum of four experiments (two replicates of two independent cultures) was performed. The relative transcript amounts were determined using 16S rRNA as the reference gene [(CtGene of interest - Ct16S) = ΔCt value].

### Biofilm/Adhesion Quantification

Biofilm formation was determined using the crystal violet (CV) assay. Briefly, overnight bacterial culture, grown in various conditions, were normalized at OD_600_ = 0.02 and incubated in triplicates (200 μl/well) in a 96-well round bottom plate for 16 h at 37°C. After quantification of their OD_600nm_ in a plate reader, planktonic cells were removed and attached cells were washed twice with sterile distilled water. A 200 μl of CV solution (1%) was added for 20 min, then the plates were washed twice, air dried, and biofilm/cell bound CV was dissolved in 96% ethanol for 10–15 min at room temperature. Finally, 100 μl of the solubilized CV were transferred to a new 96-well flat bottom plate containing 100 μl of distilled water and biofilm formation was quantified at 550 nm in a plate reader (SS Read 200) using 96% ethanol in water as the blank. The adhesion index was calculated as OD_550_ (CV)/OD_600_ (planktonic culture). Since normalization of biofilm formation to the OD_600nm_ of the planktonic cultures might lead to incorrect estimation of the actual number of bacterial cells, due to different cell morphology or production of different extracellular polysaccharide (EPS) amounts, we also determined the biofilm/planktonic cells ratio by viable counts. These experiments confirmed in full the data obtained using OD_600nm_ for normalization (data not shown).

### Motility Assay

Bacterial cells were grown overnight in YESCA growth media, with or without 8 μg/ml 6-MP, 0.2% glucose, or both, and normalized to an OD_600_ = 1. For each culture, 3 μl were spotted at the center of a motility agar plate in the same growth medium used for overnight cultures, supplemented with 0.3% agar. LF82 showed very active motility, resulting in complete colonization of the agar plate after an overnight growth. Reduction of incubation time to 8 h at 37°C allowed a more precise measurement of the bacterial halo diameters.

### Total EPS Isolation and Quantification, and Cellulose Determination

For EPS preparation, 50–100 ml of overnight cultures of bacteria grown in different conditions were centrifuged for 25 min at 5,000 rpm and resuspended in sterile PBS to an OD_600_ = 20. Normalized sample was washed twice in PBS 1×, resuspended in 1 ml of freshly made 2% EDTA pH 8.0 (in H_2_O) and incubated for 4 h at 4°C with slow orbital agitation. Before and after incubation with 2% EDTA, 10 μl of bacterial suspensions were serially diluted and plated on LB agar plates to verify the effect of EDTA treatment on cell survival. After a 4 h incubation in 2% EDTA, the suspension was centrifuged for 25 min at 5,000 rpm, and the supernatant was collected and filtered at 0.45 μm. Total sugar quantification was performed using the phenol-H_2_SO_4_ determination method (Rossi et al., 2014). Assessment of cellulose production was performed on solid media supplemented with the fluorescent dye Calcofluor (CF): overnight cultures adjusted to an OD_600_ = 0.5 and spotted on media supplemented with 0.005% CF after autoclaving. Bacteria were grown for 20 h at 37°C; phenotypes were better detectable after a further 24–48 h incubation at 4°C.

### Statistical Analysis

All experiments were performed at least three times. Statistical analysis was performed with Prism 5 software (GraphPad Software, La Jolla, CA, United States). Student’s *t*-tests for unpaired or paired samples were used to evaluate differences.

## Results

### 6-Mercaptopurine and Glucose Drastically Reduce the Invasion of Intestinal Epithelial Cells and Phagocytosis of AIEC-LF82 Strain

**FIGURE 1:**
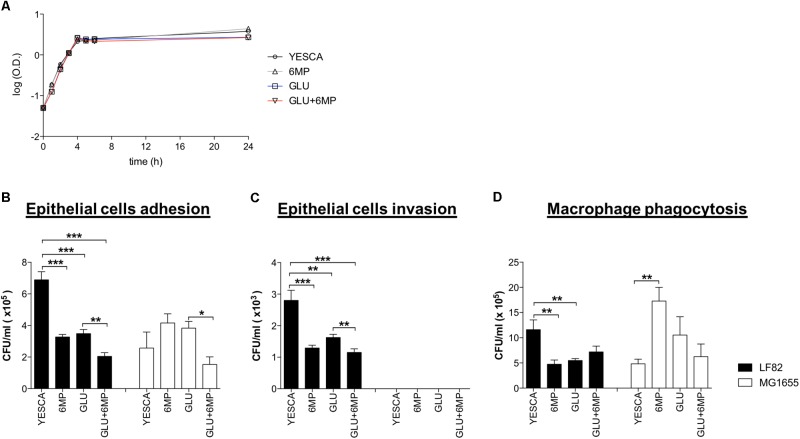
Adhesion and internalization of AIEC-LF82 and non-pathogenic MG1655 strains within intestinal epithelial cells and human monocyte-derived macrophages. **(A)** Growth curve of LF82 strain grown at 37°C in YESCA medium supplemented with 6-mercaptopurine (6-MP, 2 μg/ml), glucose (GLU, 0.2%), or both (GLU + 6-MP). **(B,C)** HT29 intestinal epithelial cell lines were infected with LF82 (black bars) or MG1655 (white bars) strains previously growth in YESCA medium supplemented with 6-MP (2 μg/ml), glucose (GLU, 0.2%), or both (GLU + 6-MP). The number of adhered **(B)** or internalized **(C)** bacteria, to assess adhesion and invasion, respectively, was determined as described in Section “Materials and Methods.” **(D)** Human MDM from healthy donors were infected with LF82 (black bars) or MG1655 (white bars) strains previously growth in the presence of 6-MP (2 μg/ml), glucose (GLU, 0.2%), or both (GLU + 6-MP). The numbers of bacteria internalized within macrophages were determined at 1 h post-infection. Results were expressed as numbers of CFU/ml and represented as mean ± SEM of triplicate experiments. Data were analyzed by Student’s *t*-test: ^∗^*p* < 0.05, ^∗∗^*p* < 0.01, ^∗∗∗^*p* < 0.001.

In order to assess whether 6-MP could affect AIEC virulence, we performed human cell adhesion and invasion assays using the CD-associated AIEC strain LF82. LF82 was grown overnight in the peptone-based, complex YESCA medium (see section “Materials and Methods”); this medium, in addition to providing a variety of carbon and nitrogen sources similar to those available in the host, can promote efficient production of several adhesion factors in *E. coli* (Hammar et al., 1995; Rossi et al., 2014). When required, 6-MP was added at 2 μg/ml. At this concentration, 6-MP does not affect LF82 growth rate (**Figure 1A**), which is inhibited, albeit partially, even at 6-MP concentrations well below the MIC of 64 μg/ml (Supplementary Figure 1).

Moreover, we grew LF82 in YESCA medium supplemented with 0.2% glucose, a concentration at which AIEC can be transiently exposed in the ileum, in order to evaluate the importance of glucose sensing, and inhibition of cAMP biosynthesis, on AIEC virulence. Finally, to assess the combined inhibition of c-di-GMP and cAMP signaling on LF82 pathogenesis, we grew it in the presence of both 6-MP and glucose. LF82 viability and growth rate were identical in all growth conditions tested (**Figure 1A**).

LF82 was grown overnight in different growth conditions, washed and normalized to the same bacterial concentrations in RPMI, prior to their incubation with human cells. Thus, bacteria were exposed to different growth media as a pre-conditioning step only, while adhesion and invasion assays were only performed in the RPMI medium. Neither intestinal epithelial cells HT29 nor human MDM were thus ever exposed to 6-MP in any of our experiments. The *E. coli* laboratory strain MG1655, grown in the same variety of conditions as LF82, was used as a non-pathogenic control in adhesion, invasion, and phagocytosis experiments.

Our data confirmed the higher proficiency of LF82 strain to adhere to intestinal epithelial cells compared to non-pathogenic strain MG1655 (Martin et al., 2004; **Figure 1B**). Notably, for bacteria pre-conditioned with 6-MP, we observed a significant reduction in the number of epithelial-adhering bacteria for LF82 (2.1-fold reduction) strain, but not for MG1655. Interestingly, the adhesion capacity of LF82 was also reduced by roughly twofold in the presence of glucose, and even further (3.37-fold in comparison to YESCA medium) when cells had been grown overnight in the presence of both 0.2% glucose and 2 μg/ml 6-MP (**Figure 1B**). On the contrary, attachment of *E. coli* MG1655 to epithelial cells was unaffected, or even slightly increased, for cells pre-grown in the presence of either 6-MP or glucose, thus suggesting that both these molecules can target the production of AIEC-specific adhesion determinants, although their combination resulted in impaired cell adhesion also in MG1655 (**Figure 1B**).

LF82 cells grown overnight in the presence of either 2 μg/ml 6-MP or 0.2% glucose showed reduced invasion of HT29 cells; addition of both glucose and 6-MP to YESCA medium showed an additive effect, resulting in further reduction of cell invasion (**Figure 1C**). These results mirror the outcome of adhesion assays, strongly suggesting that decrease in invasion is simply a consequence of reduced adhesion: indeed, the ratio of internalized/adhered LF82 cells was very similar in all cases (data not shown). Unlike LF82, the *E. coli* MG1655 laboratory strain failed to invade epithelial cells in any of the conditions tested, consistent with its non-pathogenic nature (**Figure 1C**).

**FIGURE 2:**
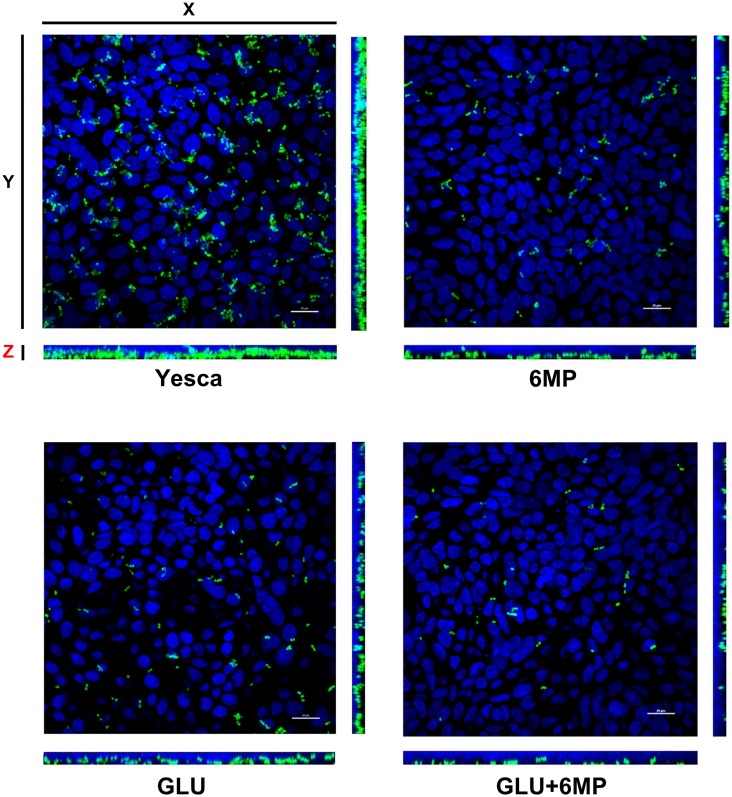
Maximum projection of Z-stack sections, obtained by confocal microscopy, of HT29 cell monolayers incubated with AIEC LF82. LF82 strain was pre-grown overnight either in YESCA medium (YESCA) or in YESCA supplemented with 2 μg/ml 6-mercaptopurine (6-MP), 0.2% glucose (GLU), or both (GLU + 6-MP) prior to a 3-h incubation with epithelial cells in RPMI medium. Cell nuclei (stained by DAPI) are shown in blue and LF82 in green. Scale bar: 20 μm. The main picture shows the view from the top, with the side views (YZ – right) and (XZ – bottom) showing sagittal sections of the infected monolayers.

To confirm the results obtained by bacterial cell counting (**Figure 1**), we also determined adhesion to human epithelial cells by LF82 pre-grown in different conditions using immunofluorescence assays followed by confocal microscopy as described in Section “Materials and Methods.” Immunofluorescence assays (**Figure 2**) showed a clear decrease in attachment of LF82 pre-grown in the presence of 6-MP, glucose, or both, in agreement with quantitative assessment by cell counting. We could not detect any adhesion of the LF82 strain to the glass slide in the absence of the epithelial cell monolayer in our experimental conditions; likewise, in monolayers not grown to 100% confluence, bacterial attachment was only observed in the areas covered by epithelial cells (Supplementary Figure 2).

Interestingly, the selective inhibitory effect of 6-MP on AIEC was also observed in phagocytosis experiments using MDM (**Figure 1D**). Indeed, in the presence of either 2 μg/ml 6-MP or 0.2% glucose, we observed significantly lower number of internalized LF82 bacteria within MDM after 1 h of infection, a reduction similar to what observed in the adhesion and invasion experiments with HT29 cells. Unlike for epithelial cells, however, the combination of glucose and 6-MP did not show any synergistic effects on phagocytosis by MDM (**Figure 1D**). Neither 6-MP nor glucose significantly prevented MG1655 uptake by MDM, again suggesting inhibition of AIEC cellular processes.

Altogether, these results suggest that 6-MP selectively affects AIEC pathogenesis, by reducing its ability to adhere to intestinal epithelial cells and its uptake by macrophages, both processes able to trigger the overly inflammatory response characteristic of CD.

### Expression of Cell Adhesion Determinants at Phenotypic Levels

**FIGURE 3:**
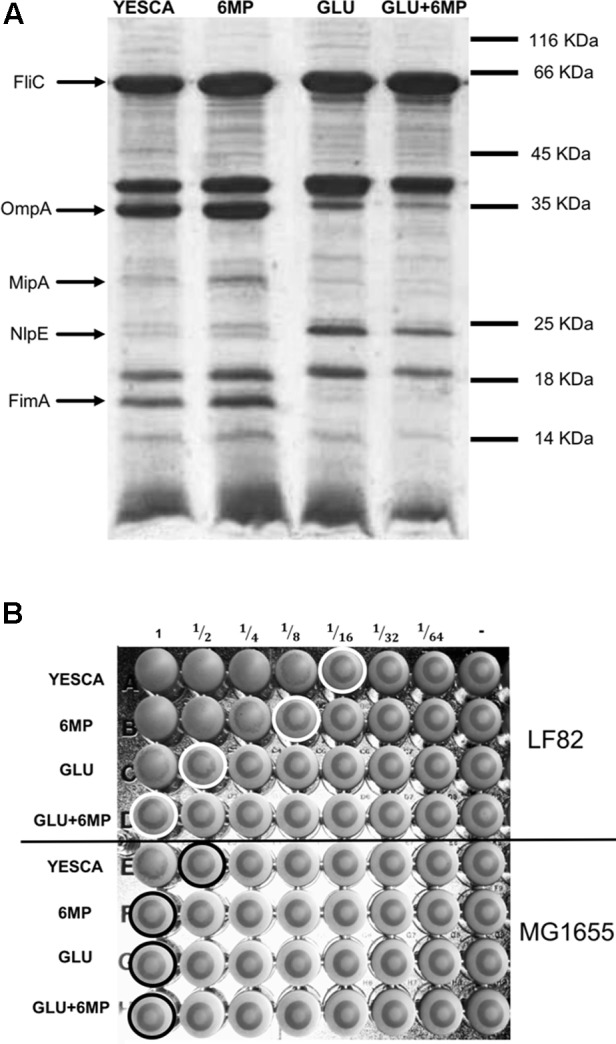
Analysis of cell surface-associated proteins and yeast agglutination assay. **(A)** SDS-PAGE of outer membrane proteins from LF82 grown in different conditions. The position of molecular weight markers is shown to the right. Bands were excised and the proteins identified with certainty by MALDI-TOF are indicated in the figure. **(B)** Ability of type 1 pili to bind D-mannose residues as determined by a yeast aggregation test. AIEC LF82 and MG1655 were growth overnight in YESCA supplemented with 6-MP (2 μg/ml), glucose (GLU, 0.2%), or both (GLU + 6-MP). A fixed amount of yeast cells (*S. cerevisiae*) suspension and decreasing concentrations of bacteria were mixed, and the loss of the ability to form homogenous aggregation was used as the read-out for impaired type 1 pili-yeast interaction. Aggregation was monitored visually, and the titer was recorded as the last dilution of bacteria giving a positive aggregation reaction as indicated by circles in the figure.

In order to identify which LF82 cell adhesion determinants might be affected by pre-growth in the presence of 6-MP, or in glucose, we observed overnight cultures grown in different conditions with transmission electron microscopy (TEM), using negative staining. TEM analysis did not show any significant changes in production of cell appendages such as flagella, a known virulence determinant in AIEC, in the different growth conditions tested (Supplementary Figure 3). To analyze further the production of possible cell adhesion factors, we isolated outer membrane and cell surface-associated proteins from LF82 overnight cultures. Cell surface-associated proteins were separated by SDS-PAGE (**Figure 3A**) and proteins differently expressed in different conditions, as determined by band intensity, as well as selected markers, were identified by MALDI-TOF. The main differences in the pattern of cell surface associated proteins were observed between cultures grown either in the absence or in the presence of glucose: MALDI-TOF analysis showed a sharp decrease in the expression of the outer membrane-associated OmpA, MipA, and FimA proteins in LF82 growing in glucose-supplemented YESCA medium, while, in contrast, the NlpE outer membrane lipoprotein was more expressed in the presence of glucose. In agreement with electron microscopy observations, flagellin, the product of the *fliC* gene, was produced at similar levels regardless of all growth conditions tested. Differential expression of the OmpA, MipA, and NlpE proteins is consistent with the lack of cAMP synthesis in glucose-supplemented media and the consequent inhibition of the cAMP/CAP protein regulatory network, which positively controls the *ompA* and *mipA* genes and represses *nlpE* (Movva et al., 1981; Tan et al., 2001; Raghavan et al., 2011).

Among the proteins downregulated in the presence of glucose, the FimA protein, the main component of type 1 fimbriae, is the most directly involved in adhesion to eukaryotic cells and it is an important determinant for AIEC virulence (Boudeau et al., 2001; Dreux et al., 2013). To verify further that production of type 1 fimbriae was affected in glucose supplementation, we performed a yeast agglutination assay, which relies on type 1 pili binding of the mannose receptors on *S. cerevisiae* cell membrane. In agreement with the results of the SDS-PAGE analysis, yeast aggregation was eightfold more efficient when LF82 cells grown in the absence of glucose were used (**Figure 3B**).

Growth in the presence of 6-MP resulted in slight changes in outer membrane-associated protein expression pattern (**Figure 3A**): however, MALDI-TOF analysis of these bands were inconclusive and did not allow us to identify any possible adhesion factor whose expression was clearly affected by 6-MP. Despite showing similar levels of FimA expression, LF82 cells grown in 6-MP-supplemented YESCA medium showed a twofold decrease in its ability to induce yeast agglutination (**Figure 3B**), in line with their reduced ability to attach to human epithelial cells (**Figure 1B**).

### 6-MP and Glucose Affect Gene Expression of Different Adhesion and Virulence Determinants in AIEC

In order to investigate the possible inhibition mechanisms of LF82 adhesion to epithelial cells and uptake by macrophages by either 6-MP or exogenous glucose, we determined the effects of either molecule on the expression of genes involved in AIEC virulence and/or encoding cell surface-associated factors, using quantitative RT-PCR (qRT-PCR). In particular, we investigated whether inhibition of type 1 pili production by glucose supplementation takes place at the transcription level, by testing *fimA* gene expression levels. In addition, we studied the *lpfA* gene, encoding the main subunit of long polar fimbriae, and the *ibeA* gene, both factors involved in epithelial cell invasion by AIEC (Barnich et al., 2003; Chassaing and Darfeuille-Michaud, 2013; Cieza et al., 2015). In addition, we also studied the expression of the *csgB* and *csgD* genes, coding respectively for the minor subunit of curli fibers (thin aggregative fimbriae) and for the CsgD protein, the master regulator of curli and cellulose production (Romling et al., 2000; Zogaj et al., 2001). Curli fibers promote internalization of *E. coli* in several eukaryotic cell lines (Gophna et al., 2001), and their expression is strongly impaired by 6-MP in *E. coli* MG1655 via inhibition of c-di-GMP biosynthesis (Antoniani et al., 2013).

**FIGURE 4:**
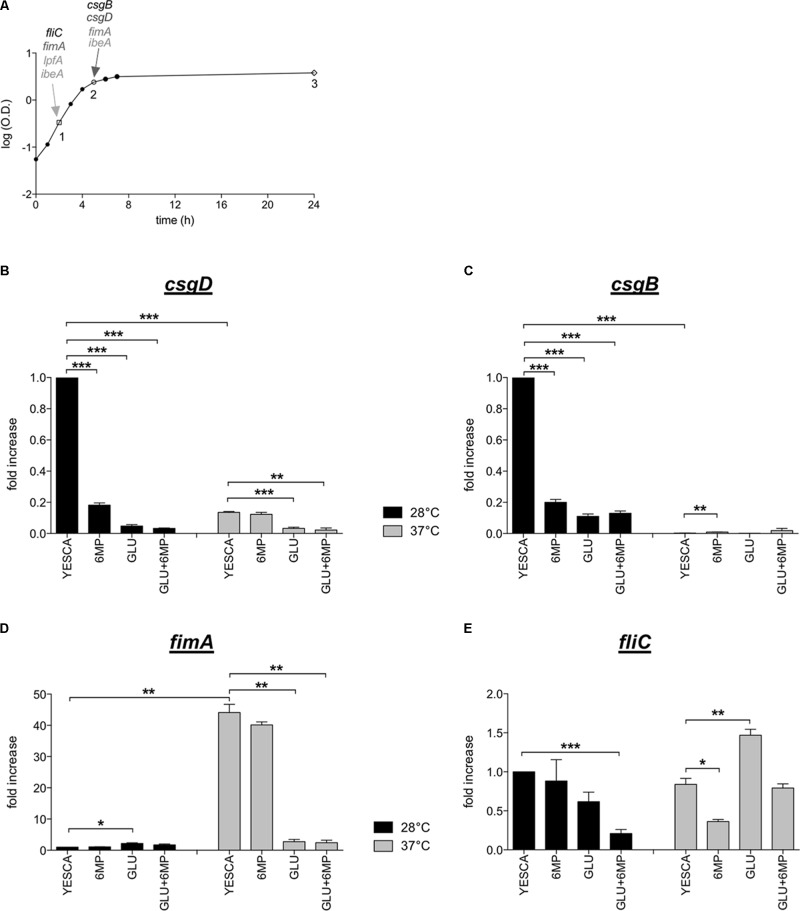
Expression of genes encoding main LF82 extracellular factors. **(A)** LF82 growth curve in YESCA medium at 37°C. A single, representative experiment is shown. Points 1, 2, and 3 indicate the time points (mid log, late log, and stationary phase) at which samples were taken for RNA extraction and determination of expression levels for the *csgD*, *csgB*, *fimA*, *fliC*, *lpfA*, and *ibeA* genes. Arrows indicate the time points at which the highest expression levels for each gene was observed, and which were used to study the effects of 6-MP and exogenous glucose. See Supplementary Figure 4 for further details. Expression of *csgD* **(B)**, *csgB* **(C)**, *fimA* **(D)**, and *fliC* **(E)** in different growth media, and at either 28°C (black bars) or 37°C (gray bars) was determined by qRT-PCR on RNA extracted at the time points indicated in panel **(A)**. For *fimA*, the time point 2 (late log) was taken. 16S RNA transcript was used as reference gene. ΔCt values between the genes of interest and 16S RNA were set at 1 for values measured in YESCA medium at 28°C, and transcript levels in other growth conditions are expressed as relative values. The ΔCt values were *csgD* = 8.4; *csgB* = 4.85; *fimA* = 11.33; *fliC* = 3.38. Data were analyzed by Student’s *t*-test: ^∗^*p* < 0.05, ^∗∗^*p* < 0.01, ^∗∗∗^*p* < 0.001.

In order to better assess possible inhibition of gene expression by either 6-MP or glucose, we performed preliminary experiments aimed at establishing the timing of maximal expression of the genes of interest. To this aim, we determined expression levels in different growth phases, namely, during exponential growth (mid log), at the onset of stationary phase (late log), and in late stationary phase (**Figure 4A** and Supplementary Figure 4). Transcription of *csg* genes is inhibited at 37°C in most enterobacteria (Romling et al., 2000), including several pathogenic *E. coli* strains (Bian et al., 2000). For this reason, in order to have a more complete assessment of gene expression levels, we carried out qRT-PCR experiments on cultures grown at both 28 and 37°C. Expression timing was very variable, with *fliC* peaking in exponential phase, *csgD* at the onset of stationary phase, and *csgB* showing highest expression levels at the onset of stationary phase in YESCA medium and in late stationary phase in YESCA supplemented with 0.2% glucose. Expression of *fimA*, *lpfA*, and *ibeA* was less stringently dependent on growth phase, and remained comparable during exponential growth and at the transition into stationary phase, while being reduced in late stationary phase (Supplementary Figure 4, and data not shown). Neither *lpfA* nor *ibeA* showed any significant changes in expression levels regardless of presence of exogenous glucose, or exposure to 6-MP (Supplementary Figure 5, and data not shown). Growth temperature did not significantly change gene expression timing, but strongly affected expression levels of *csg* and *fim* genes (**Figure 4**). In particular, transcription of *csgB*, the first gene of the *csgBAC* operon encoding the curli fibers structural subunits, was not detectable in YESCA medium at 37°C (**Figure 4C**). Both 6-MP and glucose supplementation strongly inhibited transcription of both *csgD* and *csgB* at 28°C, consistent with inhibition of c-di-GMP by 6-MP and of cAMP by glucose, respectively. Indeed, both signal molecules are necessary for full activation of the *csgD* gene (Zheng et al., 2004; Sommerfeldt et al., 2009), and, in turn, of the *csgBAC* operon.

Opposite to *csg* genes, the *fimA* and the *lpfA* genes are more efficiently transcribed (almost 50-fold) at 37°C (**Figure 4D** and Supplementary Figure 4), suggesting that the production of curli and fimbrial structures is inversely regulated by the temperature, allowing prevalent production of either adhesion factor in different environmental conditions. 6-MP does not significantly affect *fimA* transcription, which is in contrast strongly impaired in glucose-supplemented YESCA medium (**Figure 4D**), in agreement with SDS-PAGE analysis and yeast agglutination assays (**Figure 3**). Thus, presence of exogenous glucose strongly impairs transcription of both curli and type 1 pili, arguably the two main adhesion factors in *E. coli*, consistent with a reduced interaction with eukaryotic cells of LF82 grown overnight in glucose-supplemented medium (**Figure 1B**).

In the laboratory strain MG1655, *fliC* expression is downregulated in glucose-supplemented media, coherent with its cAMP activation via the class I flagellar regulator FlhDC (Zhao et al., 2007) (Supplementary Table 2). Conversely, in the LF82 strain, *fliC* expression was slightly increased in the presence of 0.2% glucose (**Figure 4E**), consistent with the results of TEM observations and SDS-PAGE analysis showing no inhibition of flagellar production in glucose-supplemented medium (Supplementary Figure 3 and **Figure 3A**). Our results strongly suggest loss of flagellar regulation by the cAMP/CAP system in AIEC, possibly as part of a pathoadaptive mechanism. Exposure to 6-MP resulted in a weak (ca. twofold), albeit reproducible, downregulation of *fliC* transcription, particularly at 37°C (**Figure 4E**).

### 6-MP Inhibits Both Biofilm Formation and Flagellar Motility in LF82

Downregulation of *csg* gene expression in LF82 confirms our previous results in MG1655 (Antoniani et al., 2013), strongly suggesting inhibition of c-di-GMP by 6-MP. However, inhibition of *csgD* gene expression by 6-MP is not detectable at 37°C (**Figure 4B**), thus making unclear whether, at this temperature, inhibition of c-di-GMP-dependent synthesis is the main mechanism of action of 6-MP.

As direct measurement of c-di-GMP in bacterial cell is not straightforward, due to its extremely low intracellular concentrations, we tested the effects of 6-MP on cell processes influenced by c-di-GMP, such as biofilm formation, EPS production and flagellar motility. In order to verify the effects of c-di-GMP on such processes in AIEC, we transformed LF82 with plasmids carrying either the *adrA* gene (encoding a diguanylate cyclase), or the *dosP* gene (encoding a c-di-GMP phosphodiesterase), in order to perturb intracellular c-di-GMP concentrations. qRT-PCR experiments confirmed that both *adrA* and *dosP* were transcribed at very high levels in LF82 transformed with the respective plasmids, in YESCA medium with or without glucose supplementation (Supplementary Figure 6).

**FIGURE 5:**
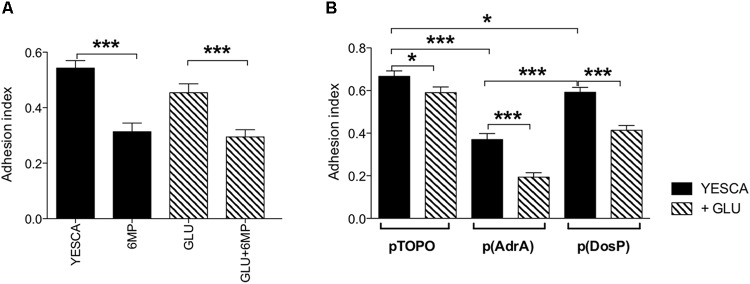
Quantitative evaluation of biofilm in crystal violet (CV) assay. **(A)** Biofilm formation by LF82 strain grown in various conditions (YESCA alone or supplemented with 2 μg/ml 6-MP, or with 0.2% glucose, or both); **(B)** biofilm formation by LF82 strains overexpressing either AdrA or DosP, or harboring the control vector pTOPO, in the presence or absence of 0.2% glucose. The adhesion index was determined after 16 h of growth at 37°C, calculated as OD_550_ (CV)/OD_600_ (planktonic culture) and expressed as mean ± SEM of triplicate experiments. Data were analyzed by Student’s *t*-test: ^∗^*p* < 0.05, ^∗∗^*p* < 0.01, ^∗∗∗^*p* < 0.001.

**FIGURE 6:**
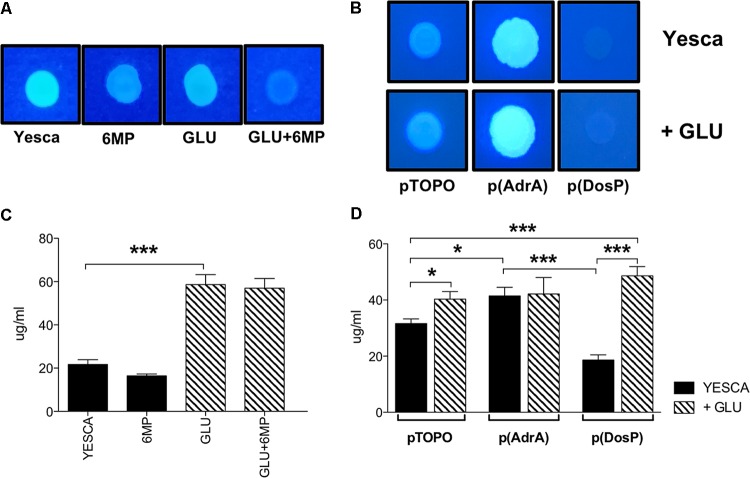
Cellulose and extracellular polysaccharide production of LF82. Cellulose visualization on Calcofluor-supplemented media. **(A)** LF82 was grown at 37°C for 24 h in YESCA supplemented with 8 μg/ml 6-MP, or 0.2% glucose, or both; **(B)** LF82 strains overexpressing either AdrA or DosP, or harboring the control vector pTOPO, were grown at 37°C for 24 h in YESCA medium in the presence or absence of 0.2% glucose. Determination of overall extracellular polysaccharide production. **(C)** LF82 grown in YESCA, alone or supplemented with 2 μg/ml 6-MP, or 0.2% glucose, or both **(D)** LF82 strains overexpressing either AdrA or DosP, or harboring the control vector pTOPO, in the presence or absence of 0.2% glucose. Data were analyzed by Student’s *t*-test: ^∗^*p* < 0.05, ^∗∗^*p* < 0.01, ^∗∗∗^*p* < 0.001.

LF82 biofilm formation on polypropylene microtiter plates was only very slightly affected by the presence of exogenous glucose (**Figure 5**), suggesting that type 1 pili, whose production is inhibited by glucose (**Figure 4D**), do not play a significant role in this process. In contrast, biofilm formation was impaired, weakly but significantly, by 6-MP (**Figure 5A**), consistent with possible c-di-GMP inhibition (Romling et al., 2013); indeed, overexpression of the c-di-GMP phosphodiesterase DosP led to a similar result. However, AdrA overexpression resulted in an even stronger reduction in LF82 adhesion, suggesting that an increase in intracellular c-di-GMP can also negatively affect biofilm formation (**Figure 5B**). This apparently counterintuitive result might depend on AdrA-dependent stimulation of cellulose production, as this EPS, while being a positive determinant for cell adhesion, can also impair adhesion to solid surfaces when overproduced (Gualdi et al., 2008). Indeed, determination of cellulose production on Calcofluor-supplemented plates clearly shows that both treatment with 6-MP and DosP overexpression impair cellulose production, which is, conversely, strongly induced by AdrA (**Figures 6A,B**). As cellulose production is dependent on c-di-GMP production (**Figure 6B**; Zogaj et al., 2001; Da Re and Ghigo, 2006) this result would suggest that 6-MP can target c-di-GMP signaling in AIEC grown at 37°C.

In terms of overall EPS production, however, availability of extracellular glucose, rather than c-di-GMP, appears to play the main role (**Figure 6C**), likely by increasing intracellular UDP-glucose concentrations available for EPS biosynthesis. Indeed, while overexpression of either AdrA or DosP significantly impacts total EPS production in LF82 grown in YESCA medium, addition of glucose completely supersedes their effects (**Figure 6D**).

**FIGURE 7:**
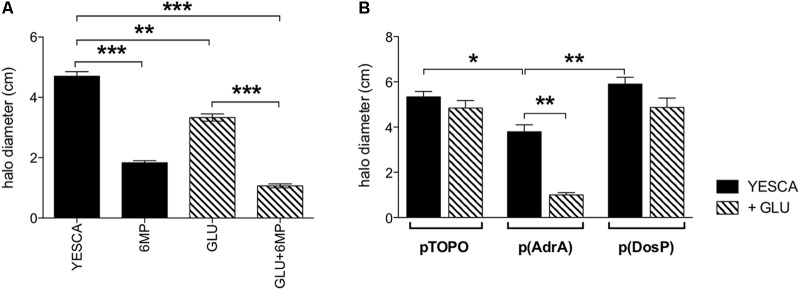
Swimming motility of LF82 strain. Flagellar motility assays. **(A)** LF82 in YESCA/0.3% agar medium supplemented with 6-MP (8 μg/ml), glucose (0.2%), alone or both (GLU + 6-MP). **(B)** LF82 strains overexpressing either AdrA or DosP, or harboring the control vector pTOPO, in YESCA medium, alone or supplemented with glucose (0.2%). Results were expressed as the halo diameter (cm) and represented as mean ± SEM of triplicate experiments. Data were analyzed by Student’s *t*-test: ^∗^*p* < 0.05, ^∗∗^*p* < 0.01, ^∗∗∗^*p* < 0.001.

Finally, we tested LF82 swimming motility, a process negatively regulated by c-di-GMP in enterobacteria (Romling et al., 2013), in the presence of 6-MP and in LF82 strains overexpressing AdrA and DosP. In line with an increase of intracellular c-di-GMP concentrations, AdrA overexpression resulted in a clear reduction in swimming motility, particularly in the presence of glucose (**Figure 7B**). Unexpectedly, however, treatment of LF82 with 6-MP also resulted in a sharp decrease in motility (**Figure 7A**), inconsistent with inhibition of c-di-GMP production. Addition of exogenous glucose also negatively affects motility, albeit at a lesser extent than 6-MP: since glucose does not affect flagellar gene expression in LF82 (**Figure 4E**), this inhibition is likely to take place via increased EPS production (**Figure 6C**), known to interfere with flagellar motility (Zorraquino et al., 2013).

## Discussion

6-Mercaptopurines, in particular the pro-drug azathioprine, are widely used as immunomodulators in the treatment of CD. Azathioprine, upon metabolic activation in the intestinal mucosa, can block T-cell activation and proliferative processes in macrophages and gut epithelial cells through inhibition of Rac1 and Rac2 proteins (Tiede et al., 2003; Marinkovic et al., 2014). In addition to its anti-inflammatory properties, 6-MP have also been shown to possess antimicrobial activity against *M. paratuberculosis* (Shin and Collins, 2008) and *Campylobacter jejuni* (Liu et al., 2017), and to inhibit c-di-GMP synthesis and cell adhesion in *E. coli* (Antoniani et al., 2013). In this work, we investigated whether 6-MP could modulate bacterial virulence in AIEC, an important pathobiont strongly correlated with CD, via inhibition of c-di-GMP signaling pathways, thus contributing to its anti-inflammation activity.

To this aim, we have carried out experiments testing the ability of 6-MP to inhibit epithelial cell adhesion and invasion by the AIEC strain LF82, important pathogenic processes directly involved in the onset of chronic inflammation in CD (Eaves-Pyles et al., 2008). In order to clearly distinguish between effects on bacterial virulence and on inhibition of Rac1/Rac2 in human cells, LF82 cultures were treated with 6-MP exclusively prior to incubation with epithelial cells, which were thus never exposed to 6-MP in our experiments. 6-MP concentrations used in these experiments did not perturb LF82 growth rate or total biomass in overnight cultures (**Figure 1A** and Supplementary Figure 1), indicating that neither viability nor the overall physiological state of the bacterium were affected, and were well below therapeutic doses administered during CD treatment (Pearson et al., 1995). Our experiments clearly demonstrate that pre-conditioning of LF82 overnight cultures with 6-MP was sufficient to impair its ability to adhere (**Figures 1B**, **2**), and consequently to invade (**Figure 1C**), epithelial cells. Another important virulence mechanism described for AIEC is its ability to survive and replicate within macrophages (Glasser et al., 2001). Our results show that uptake of 6-MP-treated LF82 by human MDMs was likewise impaired (**Figure 1D**), strongly suggesting that exposure of AIEC to 6-MP can induce a long-lasting inhibition of its cell adhesion determinants involved in interaction with different human cell types. Our results strongly demonstrate that 6-MP can inhibit adhesion to epithelial cells, and consequently their invasion, by AIEC, and impair its uptake by macrophages, exclusively targeting bacterial cell processes. As internalization into human epithelial cells, and survival in macrophages, represent strong triggers for CD inflammation (Eaves-Pyles et al., 2008; Bringer et al., 2012), inhibition of bacterial adhesion and phagocytosis might play a crucial role in anti-inflammatory activity of 6-MP. At the same time, however, reduction of AIEC uptake might result in reduced clearance of these bacteria in CD patients treated with 6-MP, which in the long term could undermine the beneficial effects of these drugs.

Unlike for LF82, exposure to 6-MP of *E. coli* MG1655 did not impair its ability either to attach to epithelial cells or to be phagocytized by human macrophages (**Figure 1**), suggesting that interaction between MG1655 and human cells might rely on a different set of cell adhesion factors, consistent with the non-pathogenic nature of this bacterium. Differences in cell adhesion regulation between AIEC and non-pathogenic *E. coli* are also highlighted by the effects of extracellular glucose on interaction with human cells: indeed, overnight growth in glucose-supplemented medium resulted in opposite effects in LF82 and MG1655 strains, somehow mirroring exposure to 6-MP (**Figure 1**). We hypothesized that the modulation of adhesion determinants in AIEC upon exposure to 6-MP and/or glucose might be due to the impairment of cell signaling pathways specific for LF82.

In *E. coli*, availability of exogenous glucose affects biosynthesis of the nucleotide-derived signal molecules cAMP. Due to its ability to inhibit *de novo* purine biosynthesis (Ha et al., 1990), we hypothesized that 6-MP might also affect cAMP production in LF82. However, at the concentrations used in our experiments, 6-MP does not affect cAMP-dependent regulation in LF82 as indicated by lack of differential expression in cAMP-dependent outer membrane proteins (**Figure 3A**), and had a different impact to glucose on *fimA* expression (**Figures 3A**, **4D**), thus suggesting that the two molecules target different signaling pathways.

Glucose inhibition of epithelial cell adhesion by AIEC, and its uptake by macrophages, possibly via inhibition of *fimA* expression, indicates that, unlike for other pathogenic bacteria (Munson, 2013; Daddaoua et al., 2014; Rossi et al., 2016), sensing of exogenous glucose availability would dampen AIEC virulence. This observation would be consistent with the ability of AIEC to cause local infection and inflammation, rather than extending to the more glucose-rich interstitial tissues and to the bloodstream.

Both 6-MP and exogenous glucose strongly downregulate transcription of the curli-related *csgB* and *csgD* genes, in line with inhibition of c-di-GMP and cAMP, respectively. Curli fibers can promote *E. coli* internalization in human epithelial cells (Gophna et al., 2001); however, in LF82, the *csgD* regulatory gene, and even more so the *csgB* gene, encoding the minor structural subunit of curli, were strongly downregulated at 37°C (**Figures 4B,C**), thus suggesting that curli do not play a role in adhesion/invasion processes in AIEC. In contrast, cellulose production was clearly detectable also at 37°C, and was strongly inhibited by 6-MP, but not by exogenous glucose (**Figure 6A**), consistent with its strong dependence on c-di-GMP (**Figure 6B**). Remarkably, cellulose can promote adhesion to epithelial cells in enterobacteria (Monteiro et al., 2009), possibly suggesting that 6-MP inhibition of LF82 adhesion to human cells might be mediated, at least in part, by reduced cellulose production. Notably, however, while cellulose is strictly dependent on c-di-GMP, overall EPS production is more affected by exogenous glucose (**Figures 6C,D**), probably via stimulation of the EPS biosynthetic pathways by increased glucose availability.

In addition to cellulose biosynthesis, c-di-GMP strongly impacts biofilm formation and cell motility, which, in turn, can affect virulence and interaction with host cells in many bacteria. As already observed for cellulose and EPS production, 6-MP and exogenous glucose impacted biofilm formation in different ways: 6-MP led to a significant reduction of biofilm formation, which conversely showed little or no inhibition by glucose (**Figure 5**). Again, this result would point to the inhibition of c-di-GMP biosynthesis by 6-MP, as also observed with DosP overexpression (**Figure 5B**), and in line with the role of this signal molecule in biofilm formation. Although biofilm formation was even more strongly affected by AdrA overexpression (**Figure 5B**), this effect might be ascribed to cellulose overproduction, which is known to negatively affect adhesion to solid surfaces (Gualdi et al., 2008).

Surprisingly, LF82 treated with 6-MP displayed a sharp decrease in swimming motility, inconsistent with c-di-GMP inhibition (**Figure 7**). This result strongly suggests that, in addition to c-di-GMP inhibition, 6-MP is able to interfere with cell signaling processes, possibly via inhibition of *de novo* purine synthesis (Ha et al., 1990), which might in turn affect nucleotide-derived signal molecules other than c-di-GMP. Inhibition of swimming motility by 6-MP might play a significant role in downplaying LF82–host cell interaction, as it was shown that non-motile AIEC *fliC* mutant strains have a significantly reduced ability to invade intestinal epithelial cells (Eaves-Pyles et al., 2008). Inhibition of flagellar motility might also be the main mechanism by which 6-MP negatively affects biofilm formation, as flagella have been shown to be necessary for initial surface colonization by several Gram-negative bacteria (Pratt and Kolter, 1998).

## Conclusion

In conclusion, our results have highlighted a novel, additional role for 6-MP, targeting bacterial virulence rather than human cells. In particular, micromolar concentrations of 6-MP can prevent AIEC adhesion to human epithelial cells and uptake by macrophages, likely via inhibition of cellulose production and cell motility. Since 6-MP impairs c-di-GMP biosynthesis (Antoniani et al., 2013), targeting this signal molecule could be an interesting strategy to hamper AIEC virulence. Although AIEC is not the sole responsible for the onset of chronic inflammation of CD, and our study did not tackle the full complexity of this disease, our results allow us to propose that targeting virulence factors of CD-related bacterial pathobiont species might represent a valuable therapeutic strategy in CD remission. Indeed, while 6-MP remain an integral part of disease management in IBD patients, it has been reported that a small, albeit clear, association exists between non-melanoma skin cancer and lymphoma with long-term thiopurines usage (Beaugerie et al., 2009; Peyrin-Biroulet et al., 2011), as well as a moderate-to-severe relapse rate after thiopurines withdrawal (Kennedy et al., 2014). Thus, novel molecules targeting virulence factors of pathobionts, while leaving not only eukaryotic cells, but also commensal and beneficial microorganisms unaffected, would be an extremely useful therapeutic strategy in treatment of CD patients.

## Ethics Statement

In our manuscript, we report experiments performed with human cells obtained from buffy coat from healthy anonymous donors. According to the Italian laws, the donors signed an informed consent form, allowing utilization of blood-derived cells for basic research studies. No further authorization from the Ethical committee is necessary for such studies.

## Author Contributions

MP and PL conceived and designed the experiments, contributed reagents/materials/analysis tools, analyzed the data, and wrote the paper. FM, RM, and MP performed the experiments.

## Conflict of Interest Statement

The authors declare that the research was conducted in the absence of any commercial or financial relationships that could be construed as a potential conflict of interest.

## Footnotes

1 http://www.ceinge.unina.it/
