# Supplementary material for: Phagocytosis and Epithelial Cell Invasion by Crohn’s Disease-Associated Adherent-Invasive Escherichia coli Are Inhibited by the Anti-inflammatory Drug 6-Mercaptopurine
Source: Front Microbiol. 2018 May 14;9:964. doi: 10.3389/fmicb.2018.00964 (PMC5961443; doi:10.3389/fmicb.2018.00964)
Supplement: Supplementary file 1 [file Image_1.pdf]

Supplementary Figure 1

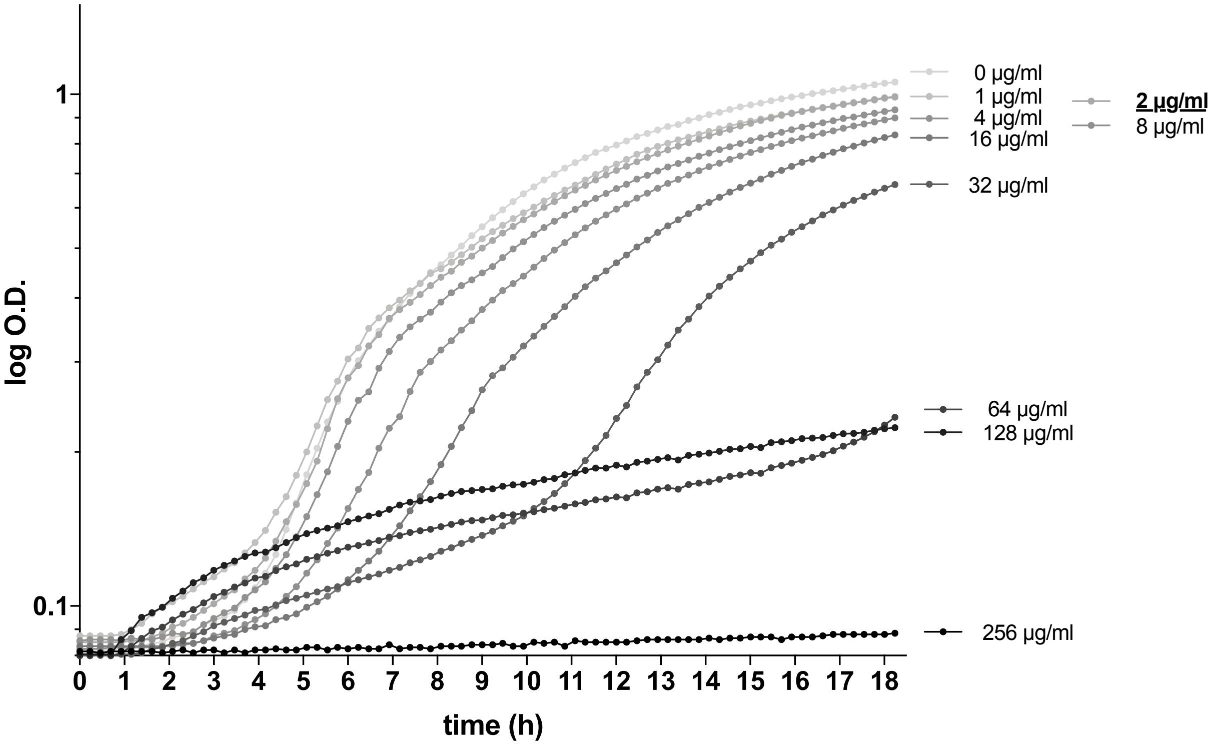

Supplementary Figure 1: Growth inhibition of 6MP on AIEC LF82.

Growth curves of LF82 in the presence of increasing concentrations of 6-MP (0 -256 µg/ml) in YESCA, in microtiter plates at 37°C, measured as OD_{600}. The concentration chosen for all subsequent experiments (**2 µg/ml**) is shown in bold.

Supplementary Figure 2

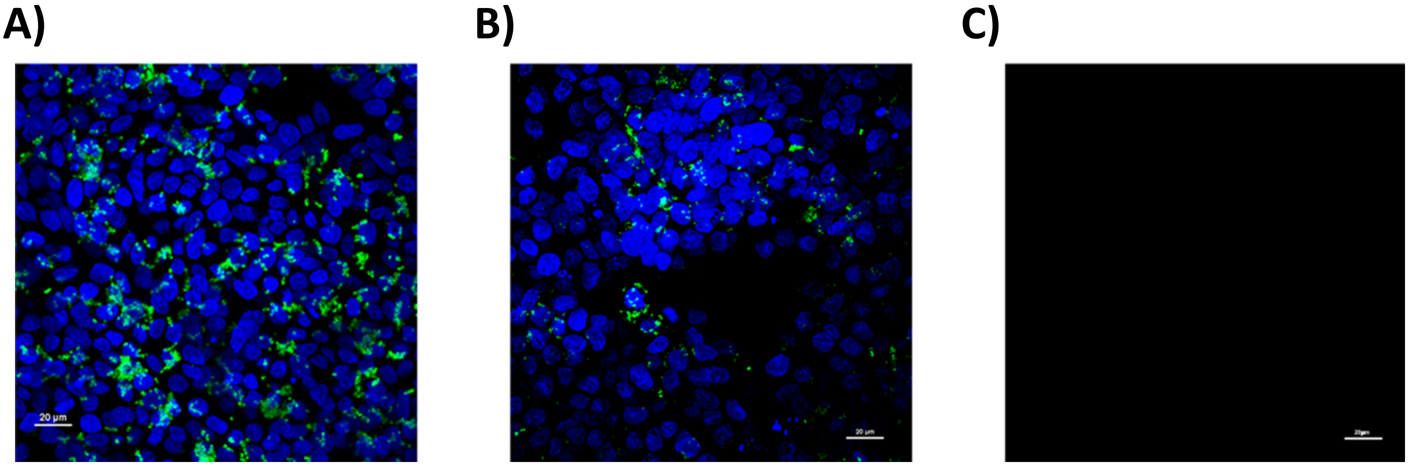

Supplementary Figure 2: Representative confocal immunofluorescence images of LF82 adhesion to the epithelial cells or glass slides.

Z-stack maximal projection of HT29 cells at 100% confluence (monolayer, A), 50% confluence (B) and coverslips (C, absence of epithelial cells) incubated with LF82 strain for 3h.

Cell nuclei (DAPI) are shown in blue and LF82 in green. Scale bar: 20µm

Supplementary Figure 3

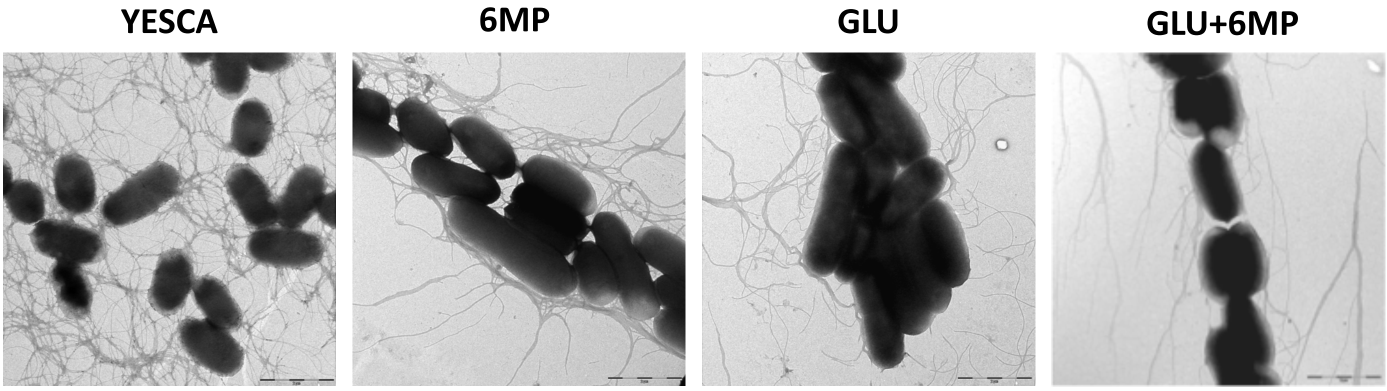

Supplementary Figure 3: Transmission electron microscope (TEM) examination of LF82.

LF82 grown in different media, as indicated, were fixed and negatively stained with 1% ammonium molybdate on carbon-Formvar copper grids. The black scale bar indicates 2 µm.

Supplementary Figure 4

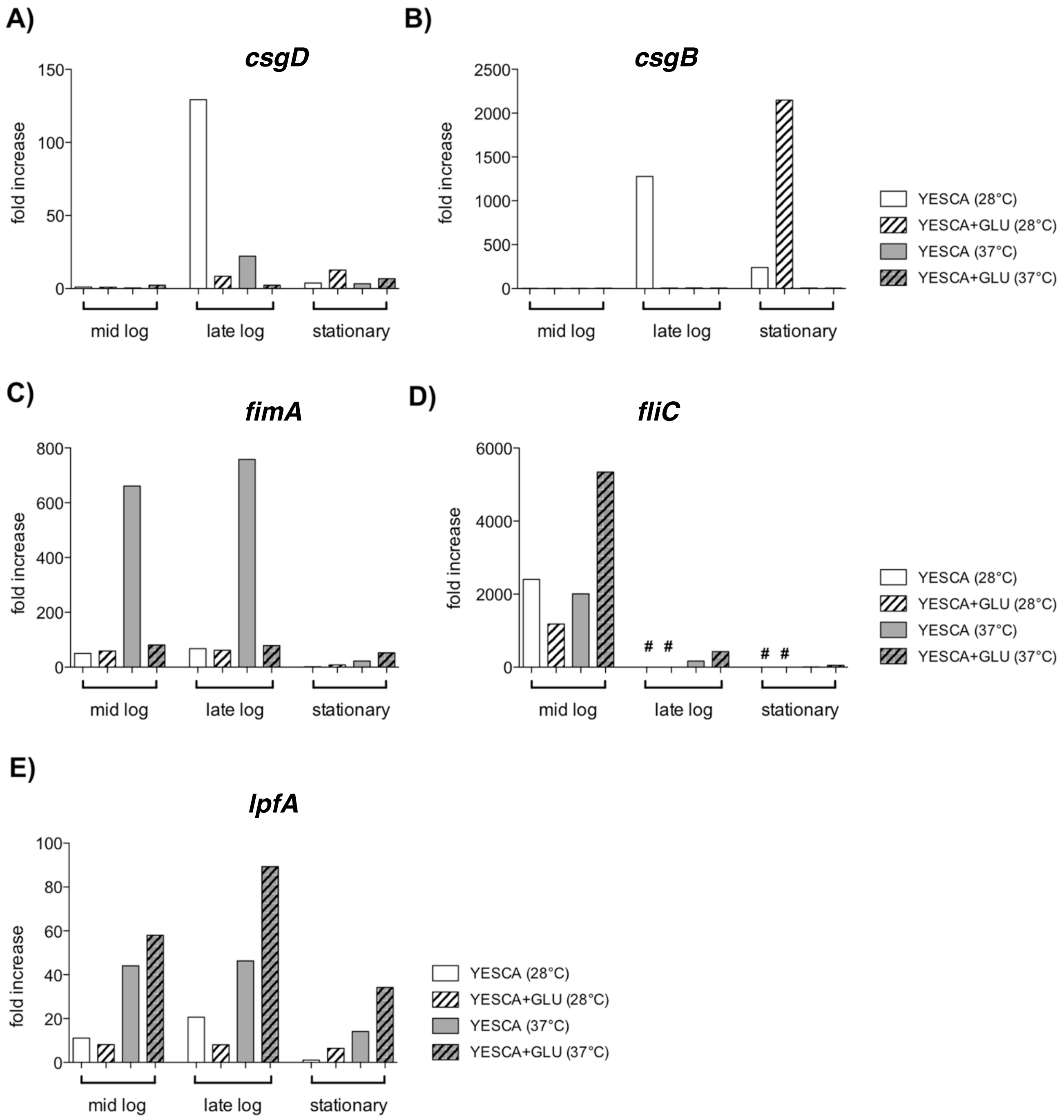

Supplementary Figure 4: Expression timing of genes encoding main LF82 extracellular factors.

Timing of *csgD* (A) *csgB* (B), *fimA* (C), *fliC* (D) and *lpfA* (E) in LF82 grown in different media, and at either 28°C (white bars) or 37°C (grey bars) was determined by qPCR on RNA extracted at the different time points (mid-log, late log, and stationary phase as reported in Figure 2A). 16S RNA transcript was used as reference gene.

*Δ*Ct values between the genes of interest and 16S RNA were set at 1 for values measured in YESCA at the time point at which expression levels for each gene was the lowest, and the transcript levels in other growth conditions are expressed as relative values.

(# not determined)

Supplementary Figure 5

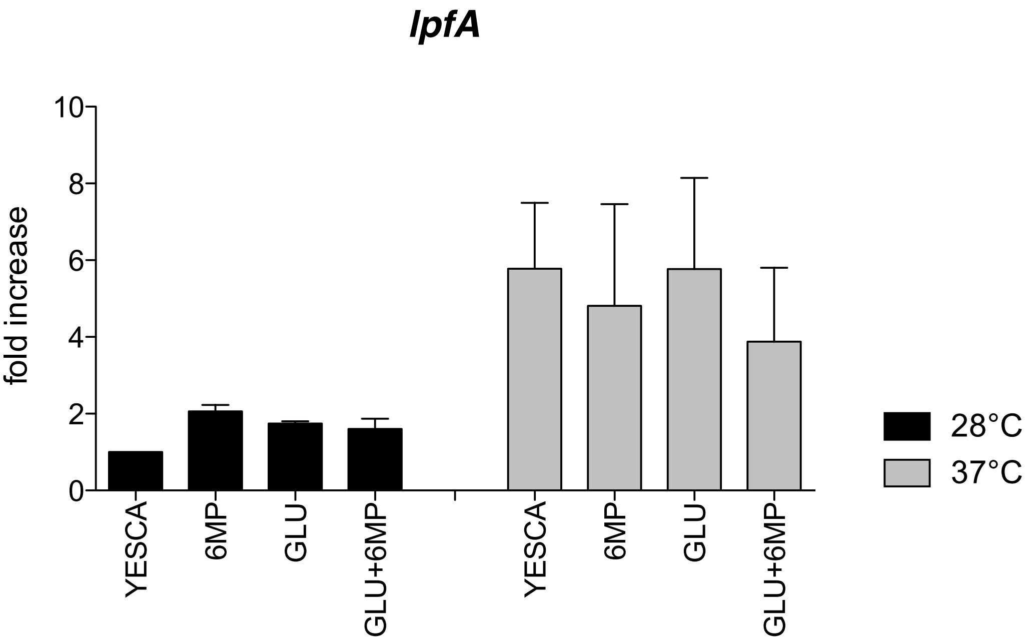

Supplementary Figure 5: Expression levels of *lpfA* gene in LF82

Expression of *lpfA* in different growth media, at either 28°C (black bars) or 37°C (grey bars) was determined by qPCR on RNA extracted at the mid log phase. 16S RNA transcript was used as reference gene.
*Δ*Ct values between *lpfA* and 16S RNA were set at 1 for values measured in YESCA medium at 28°C, and transcript levels in other growth conditions are expressed as relative values.

Supplementary Figure 6

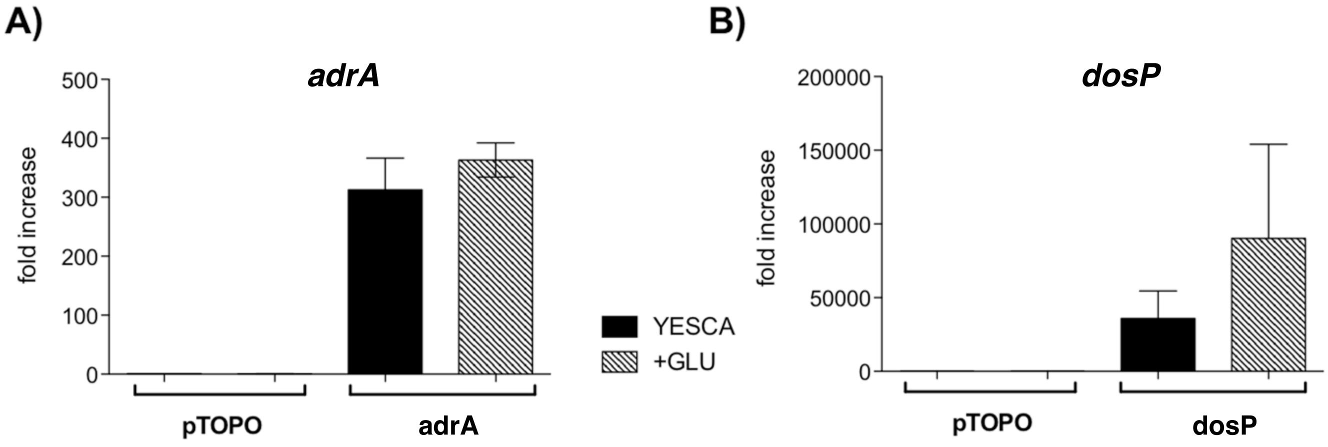

Supplementary Figure 6: Overexpression of *adrA and dosP* in LF82

Expression of *adrA* or *dosP* in YESCA (black bars) or YESCA supplemented with glucose (grey bars) was determined by qPCR on RNA from LF82 transformed either with plasmid carrying the *adrA* gene or the *dosP* gene or with the pTOPO vector.
16S RNA transcript was used as reference gene. ΔCt values between *adrA* or *dosP* and 16S RNA were set at 1 for values of LF82 harbouring the control vector (pTOPO) measured in YESCA medium, and transcript levels in other growth conditions are expressed as relative values.
ΔCt values in the LF82pTOPO strain were: *adrA=1*6.18 and *dosP*=18.9.

Supplementary Table 1: Primers used in this study.

PRIMER	SEQUENCES 5'-3'
16 S_for	TGTCGTCAGCTCGTGTCGTGA
16 S_rev	ATCCCCACCTTCCTCCGGT
*adrA*_for	GGCTGGGTCAGCTACCAG
*adrA*_rev	CGTCGGTTATACACGCCCG
*dosP*_for	CAGAGAAGCTCTGGGGATACA
*dosP*_rev	TTTTTCTCCAGCTGCAGCTCC
*fimA*_for	CGCTTGCGCAGTTGATGCAG
*fimA*_rev	CCGTCCCCAAGAAGGCAACA
*csgB*_for	CATAATTGGTCAAGCTGGGACTAA
*csgB*_rev	GCAACAACCGCCAAAAGTTT
*csgD*_for	CCCGTACCGCGACATTG
*csgD*_rev	CGTTCTTGATCCTCCATGG
*lpfA*_for	TCACTGTCGGCAACCACAG
*lpfA*_rev	GAGAACCGCTAATCCCATGT
*fliC*_for MG1655	ACAGCGTGTGCGTGAACTG
*fliC*_rev MG1655	CTGACCAGATACGCGGTCA
*fliC*_for LF82	CAACTTACAGCGTATCCGTG
*fliC*_rev LF82	CGTTCACGCCGTTGAACTG
*ibeA*_for LF82	ACCCGCTCGTAATATACCTG
*ibeA*_rev LF82	TCATTCCGCCAAAACAACC

Supplementary Table 2: Expression levels of *fliC* gene in LF82 compared to MG1655

	MG1655 (YESCA)	MG1655 (YESCA+GLU)	LF82 (YESCA)	LF82 (YESCA+GLU)
**Mid log**	1	0,047	9,043	18,515
**Late log**	0,754	0,041	0,403	1,031
**Stationary**	0,004	0,013	0,003	0,128

Expression level of *fliC* gene relative to the 16S RNA gene, used as reference (*Δ*Ct=6.55), was set at 1 for MG1655 grown in YESCA in mid log phase, at which its expression levels is the highest. Transcript levels in other conditions or in LF82 are indicated as relative fold induction values.
